# Generating a mouse model for relapsed Sonic Hedgehog medulloblastoma

**DOI:** 10.1016/j.xpro.2023.102234

**Published:** 2023-04-18

**Authors:** Allie Heller, Fang Du, Yongqiang Liu, Yijun Yang, Zeng-Jie Yang

**Affiliations:** 1 Nuclear Dynamics and Cancer Program, Fox Chase Cancer Center, Temple University Health System, Philadelphia, PA 19111, USA

**Keywords:** Cell Isolation, Cancer, Microscopy, Model Organisms, Neuroscience

## Abstract

Tumor relapse is the leading adverse prognostic factor in medulloblastoma (MB). However, there is still no established mouse model for MB relapse, impeding our efforts to develop strategies to treat relapsed MB. We present a protocol for generating a mouse model for relapsed MB using irradiation by optimizing mouse breeding and age, as well as irradiation dosage and timing. We then detail procedures for determining tumor relapse based on tumor cell trans-differentiation in MB tissue, immunohistochemistry, and tumor cell isolation.

For complete details on the use and execution of this protocol, please refer to Guo et al. (2021).^1^

## Highlights

• Strategy of generating a relapsed MB model in mice by irradiation
• Optimal irradiation dosage and age of tumor-bearing mice for MB relapse
• Determination of tumor relapse based on tumor cell trans-differentiation in MB tissue
• Detailed analysis of tumor cell proliferation and apoptosis during MB relapse

## Abstract (editor highlights)

Publisher’s note: Undertaking any experimental protocol requires adherence to local institutional guidelines for laboratory safety and ethics.

## Graphical abstract

**Figure:**
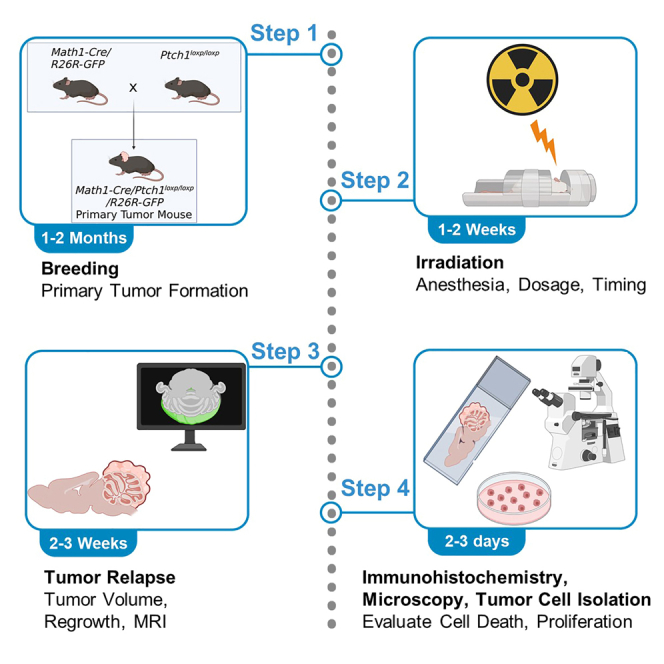

## Before you begin

### Background

Medulloblastoma (MB) is the most common type of brain tumor in children, comprising four principal groups: WNT Group, Sonic Hedgehog (SHH) Group, Group 3 and Group 4. The most adverse prognostic factor of all MB diagnoses is tumor relapse, occurring in approximately 30% of all cases, and often fatal.^2^ The functional heterogeneity within relapsed MB tumors presents significant therapeutic challenges.^3^ To recapitulate this in a mouse model, we use the conditional deletion of Patched 1 (Ptch1) in cerebellar granule neuron precursors using Math1-Cre mice, resulting in MB formation in Math1-Cre/Ptch1^loxp/loxp^ mice with 100% penetrance.^4^ By further lineage tracing and genomic sequencing, recent studies reveal that tumor cells trans-differentiate into astrocytes in relapsed MB.^1^

#### Calculate irradiation dosage/timing (formula)

Model 280 Cesium Irradiator Dose Rates:

For 3″ diameter field, no attenuation sources in: 0.862 Gy/min.

1. 4 Gy = 4 min, 38 s.
2. 2 Gy = 2 min, 19 s.
3. 1 Gy = 1 min, 10 s.
4. 0.5 Gy = 35 s.

***Note:*** Calculation of dosage rates varies for different irradiator devices. Dosage rates should be adjusted and corrected for decay according to irradiator device (e.g., Model 280 Cs137 decay factor = 0.9886 in 6 months).

#### Calculate the exposure rate

Basic Equation^5^: $\dot{X}=\dot{X_{0}}\;e^{-\frac{u}{\rho }\;\rho x}$.

a. X- exposure rate with shield in place.
b. X_0_- exposure rate without shield.
c. x- thickness of shield.
d. u/p- mass attenuation coefficient.
e. p- density of shielding material.

Based on the dose-rate and thickness of shield, the exposure rate with shield in place (X_0_) can be calculated based on the above equation.

An example of this equation is provided here (Source: Cesium-137):

 Exposure rate without shield (Cs-137): X_0_ = 180,000 R/h.

 Shield thickness (Pb):   x = 0.11 cm.

 Mass attenuation coefficient (Pb):   u/p = 0.11 cm^2^/g.

 Density of shielding material (Pb):  ρ = 11.4 g/cm^3^.

 Exposure rate with lead shield (Cs-137): Ẋ = 9.86605E-14 Gy/s.

***Note:*** The actual exposure of mouse body parts except the brain should be less than 0.008 Gy with the lead shield in place.^6^ A single dosimetry radiochromic film should be used to confirm efficient protection of lead shield from the irradiation.

### Institutional permissions

**Animals:** Math1-Cre mice, Ptch1^loxp/loxp^ mice, and R26R-GFP mice were purchased from the Jackson laboratory, and maintained in the Fox Chase Cancer Center Laboratory Animal Facility (LAF). All mice were genotyped as recommended by the Jackson Laboratory. All experiments were performed in accordance with procedures approved by the Fox Chase Cancer Center Animal Care and Use Committee (IACUC), and the Institutional Biosafety Committee (IBC).

**Irradiation:** Users of Model 280 Cesium Irradiator must be approved and granted permission by the radiation safety officer required by the NRC regulations and must be trained and tested concerning security procedures, radiation safety, emergency procedures, and proper operation of the unit.

## Key resources table

**Table:** 

REAGENT or RESOURCE	SOURCE	IDENTIFIER
**Antibodies**		

Mouse anti-S-100 (β-subunit) antibody (1:400 dilution)	Sigma-Aldrich	Cat# S2532
Goat anti-mouse IgG (H+L) secondary antibody, Alexa Fluor® 647 conjugate (1:500 dilution)	Thermo Fisher	Cat# A28181
Rabbit anti-cleaved - caspase-3 (Asp175) (5A1E) antibody (1:400 dilution)	Cell Signaling Technology	Cat# 9664
Rabbit anti-Ki67 antibody r (1:250 dilution)	Abcam	Cat# ab15580
Goat anti-rabbit IgG (H+L) secondary antibody, Alexa Fluor® 594 conjugate (1:500 dilution)	Thermo Fisher	Cat# A11012
DAPI (1:1000 dilution)	Roche Sigma	Cat# 10236276001

**Chemicals, peptides, and recombinant proteins**		

1× DPBS, calcium, magnesium	Thermo Fisher	Cat# 14040133
Normal goat serum (NGS)	Cell Signaling	Cat# 5425S
Saline solution	Thermo Fisher	Cat# R064432
Ketamine	Vedco	Cat# AHO2WWH
Xylazine	Bimdea-MTC	Cat# 1XYL0038XYL006
D-Mannitol	Sigma-Aldrich	Cat# M4125
Paraformaldehyde solution (PFA), 4% in PBS	Affymetrix	Cat# 19943
Ultrapure sucrose	Thermo Fisher	Cat# 15503022
Virkon S biocidal tablets	Fisher Scientific	Cat# NC9549979
Triton X-100	Fischer Scientific	Cat# AAA16046AE

**Experimental models: Organisms/strains**		

Mouse: Math1-Cre (breeding-age males and females)	Jackson Laboratory	Stock #: 011104
Mouse: Ptch1^loxp/loxp^ (breeding-age males and females)	Jackson Laboratory	Stock #:030494
Mouse: R26R-GFP (breeding-age males and females)	Jackson Laboratory	Stock #:004077

**Oligonucleotides**	Jackson Laboratory	

Primer	Sequence (5′ → 3′)	
Math1-Cre-F	CCG GCA GAG TTT ACA GAA GC	IDT
Math1-Cre-R	ATG TTT AGC TGG CCC AAA TG	IDT
Ptch1^loxp/loxp^-F	CCA CCA GTG ATT TCT GCT CA	IDT
Ptch1^loxp/loxp^-R	AGT ACG AGC CAT GCA AGA CC	IDT
Internal Control-F	CTA GGC CAC AGA ATT GAA AGA TCT	IDT
Internal Control-R	GTA GGT GGA AAT TCT AGC ATC ATC C	IDT
R26R-GFP-F	CGC CTA AAG AAG AGG CTG TG	IDT
R26R-GFP-R	GAA CTT CAG GGT CAG CTT GC	IDT

**Software and algorithms**		

SPSS Statistics	Sigma	Free Download

**Other**		

Nikon Eclipse Ti fluorescent microscope	Microscope Guru	Cat# 25089
Nikon SMZ1500 stereo microscope	Spach Optics	Cat# NIKON-SMZ1500
Model 280 cesium irradiator	J.L. Shepherd	N/A
Cryostat	Leica	Cat# CM3050 S
15 mL conical centrifuge tube	Fisher Scientific	Cat# 14-959-53A
Surgical scissors	Fine Science Tools	Cat# 14090-09
Dissection scissors	Fine Science Tools	Cat# 14060-09
Scalpel handle	Fine Science Tools	Cat# 10004-13
Scalpel blades	Fine Science Tools	Cat# 10023-00
Forceps	Fine Science Tools	Cat# 11252-00
Gloves	Denville	Cat# 1159G05
Deltaphase isothermal pad	Braintree Scientific	Cat# 39DP
Nutrition gel	ClearH2O	Cat# 72-06-5022
BD Insulin Syringes with BD Ultra-Fine™ Needle 8 mm × 31G 1 mL/cc	BD	Cat# 328418
Phillips Safety Lead Sheeting 1/16″ Thickness	Kemper Medical	Cat# MAR-PB-12X12-1/16
GAFChromic Film, EBT3, 8 × 10	Radiation Products Design Inc.	Cat# 115-016
CO2 tank	Airgas	Cat# CD 50
OCT compound	Fisher Scientific	Cat# 23-730-571
Epredia™ Peel-A-Way™ Disposable Embedding Molds	Fisher Scientific	Cat# 12-20
ImmEdge Hydrophobic Barrier PAP Pen (Vector Labs)	Fisher Scientific	Cat# NC9545623
Fisherbrand Superfrost Plus Microscope Slides	Fischer Scientific	Cat# 22-034-979
Fluromount G 25 ML	Fisher Scientific	Cat# 00-4958-02
Glass coverslip round 12 mm	Fisher Scientific	Cat# 12-545-80

## Materials and equipment

### Anesthetic solution

• Dilute 1 mL of 100 mg/mL Ketamine and 0.25 mL of 20 mg/mL Xylazine in 8.75 mL of saline solution.
• For a final concentration of 10 mg/mL Ketamine and 0.5 mg/mL Xylazine, total volume of 10 mL.
  ○Store solution at 20°C–25°C, stable for up to 6 months.

10% Normal Goat Serum (NGS).

• Dilute 1 mL of Normal Goat Serum in 9 mL of 1× PBS or 1× PBST for a final volume of 10 mL.
  ○Store at 2°C–8°C, stable for minimum of 1 year.

0.1% Triton X-100 in 1× Phosphate-Buffered Saline (PBST).

• Mix 100 mL of 10× PBS and 1 mL of Triton X-100 in 0.899 L of distilled H_2_O for a final volume of 1 L PBST.
  ○Store at 18°C–26°C, stable for 6 months.

4% Paraformaldehyde (PFA).

• Dilute 4 g of paraformaldehyde in 50 mL of 1× PBS.
  ○Store at 4°C.

30% Sucrose.

• Dilute 30 g of sucrose in 100 mL of 1× PBS.
  ○Filter and store solution at 4°C for up to one month.

20% Mannitol.

• Dilute 20 g of D-Mannitol in 100 mL of 1× PBS or saline solution for a final volume of 100 mL.
  ○Store solution at 20°C–25°C.

## Step-by-step method details

### Part 1: Breeding

**Timing: 4 months (for step 1)**

1. Generating Math1-Cre/Ptch1^loxP/loxP^/R26R-GFP (MPG) Mice.
  Generation of MPG mice that will develop MB in their cerebella.
  a. Cross Math1-Cre mice with Ptch1^loxp/loxp^ mice to obtain Math1-Cre/Ptch1^loxp/wt^ mice.
  b. Cross Ptch1^loxp/loxp^ mice with R26R-GFP mice to obtain Ptch1^loxp/wt^/R26R-GFP mice.
  c. Cross Math1-Cre/Ptch1^loxp/wt^ mice with Ptch1^loxp/wt^/R26R-GFP mice to obtain MPG mice.
    ***Note:*** R26R-GFP mice are not necessary for the generation of the relapse model. We use R26R-GFP mice to lineage-trace tumor cells in relapsed MB (Mao et al.^7^).
    ***Note:*** Math1-Cre/Ptch1^loxp/wt^/R26R-GFP mice obtained from the above step c crossing can be backcrossed to Ptch1^loxp/loxp^ mice to generate MPG mice. MPG mice can also be generated by intercrossing Math1-Cre/Ptch1^loxp/wt^/R26R-GFP mice.
    ***Note:*** All MPG mice develop MB in their cerebella. MPG mice may display cranial tumor signs including ataxia, hunched back and tilted head starting from 3–4 weeks of age.
    ***Note:*** To determine the optimal stage of tumor development for generating tumor relapse, MPG mice at 2 or 4 weeks of age are used for irradiation.

### Part 2: Irradiation

**Timing: 30 min (for step 2)**

**Timing: dosage dependent; ∼ < 5 min/mouse (for step 3)**

**Timing: 12 h–14 days (for step 4)**

***Note:*** A license authorizing the use of sealed sources containing radioactive material may be required to operate the irradiator. Consult the institutional irradiation safety office for the regulations.

**Figure 1:**
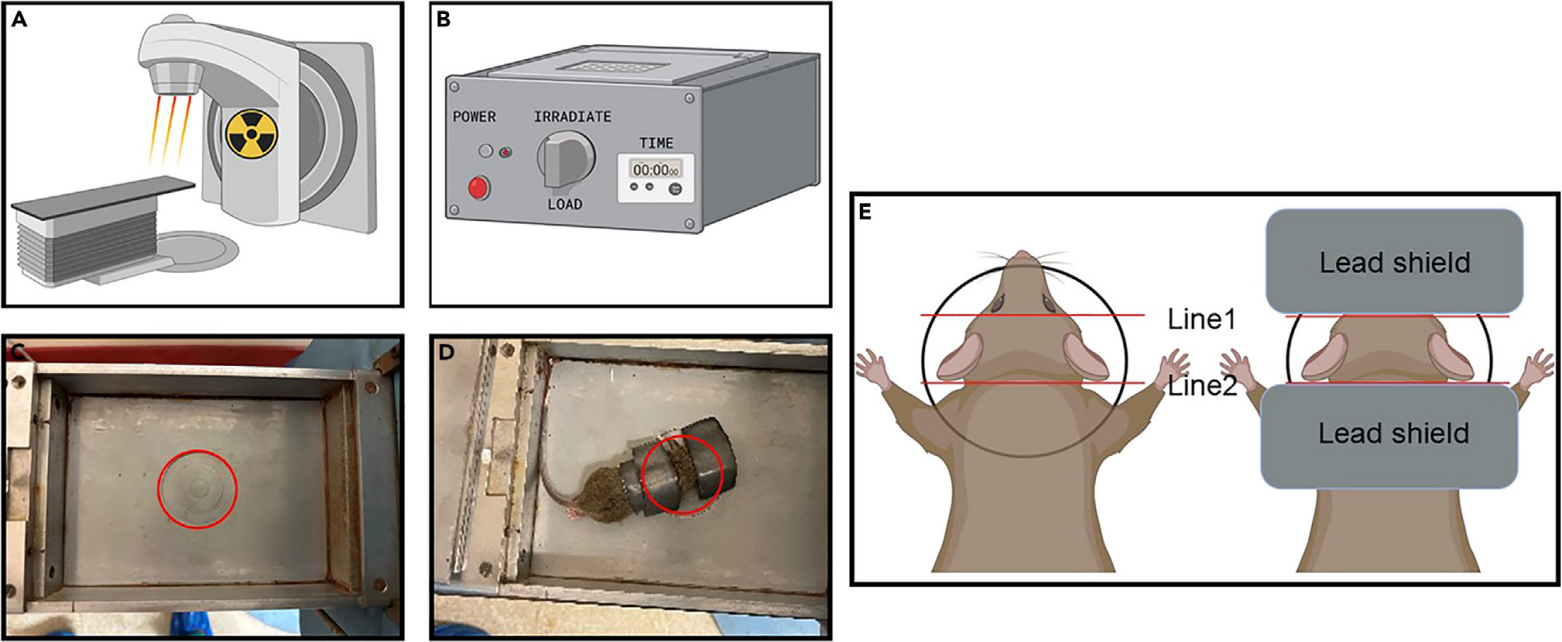
Irradiation procedure (A and B) Schematic images showing the (A) irradiator and the (B) irradiator control panel. (C) An empty leucite drawer showing where to position the mouse skull in the irradiation area (in red). (D) An MPG mouse placed in the irradiated area (in red) and covered with lead shields both around the base of the skull and above the tumor area. (E) Schematic representation to show placement of lead shield on mouse, allowing for the irradiation exposure of the cerebellar area.
    ii. Expose the cerebellum, but shield the rest of the brain with the lead cover.
      ***Note:*** The cerebellum is located between the two lines (Line 1 and Line 2 in Figure 1E). Line 1 is between the two eyes, and line 2 is aligned with the base of the skull.

2. Prepare Mice for Irradiation.
  Mice must be properly anesthetized for the irradiation procedure to minimize pain and distress, as well as movement during the procedure to ensure accurate dosages of irradiation.
  a. Prepare 10 mL Ketamine/Xylazine Anesthetic Solution.
    i. Prepare 1 mL of Ketamine (100 mg/mL) at a final concentration of 10 mg/mL.
    ii. Prepare 0.25 mL of Xylazine (20 mg/mL) at a final concentration of 0.5 mg/mL.
    iii. Mix the above with 8.75 mL of saline solution to prepare a final 10 mL anesthetic solution.
      ∗The above solutions are prepared under sterile conditions.
  b. Administer Ketamine/Xylazine via intraperitoneal (IP) injection.
    i. Measure body weight of mice using an animal weighing scale.
    ii. Inject mice with the Ketamine/Xylazine solution (10 μL/g body weight).
      ∗ Appropriate depth of anesthetizing in mice should be confirmed by verifying a lack of pain response by pinching tails (no longer than 10 min after injection of Ketamine/Xylazine solution).
      **CRITICAL:** Ketamine is a controlled substance, which should be used following the guidelines of institutional IBC.
      **CRITICAL:** Mice should be euthanized if they display signs of ketamine overdosage (respiratory depression).
  c. Position and shield mice for irradiation.
    i. Position the mouse (4 weeks of age) lying prone in a leucite drawer (As shown in Figure 1), and make sure that the cerebellum is centered inside of the circular irradiation area.

3. Perform Irradiation Procedure.
  Following approval, irradiation procedure is conducted to reduce tumor burden allowing subsequent tumor relapse.
  a. Obtain instruction and approval from the Institutional Radiation Safety Committee before using the irradiator machine.
  b. Prepare the irradiator machine as necessary to begin procedures for irradiation of mice (i.e., air tank and pressure valves).
    ***Note:*** Be sure there is an air pump to ventilate the drawer for live animals, if necessary.
  c. Remove lid and place the mouse into the Leucite drawer.
    i. Center the base of the skull inside of the circular irradiation area.
    ii. Shield the mouse brain using the lead cover, exposing the cerebellum region.
    iii. Cover the drawer with mouse inside by replacing the leucite lid.
  d. Set the irradiation time(s) and begin procedure.
  e. Remove mouse from the drawer, repeat for additional mice (steps 3–5).
    ***Note:*** To maintain a sterile condition, the leucite drawer can be wiped with a biocide solution in between each mouse irradiation.
  f. Secure irradiator after use.
    ***Note:*** If any problems or questions, contact the radiation safety office and read all operating procedures. Do not attempt any adjustment or repairs without authorization.
    **CRITICAL:** Make sure the mouse does not move at all in the drawer during the irradiation procedure. Any movement could potentially alter the irradiation dosage that the mouse cerebellum has actually received.

4. Post-Irradiation Care.
  Mice must receive the proper post-irradiation care for their optimal survival throughout the experiment.
  a. Post irradiation, place mice on top of warm isothermal pad until they regain upright posture and walk normally.
  b. Return all of the irradiated mice to a sterilized cage, with adequate food and water.
  c. Routinely monitor mice twice daily or more often if poor health is observed (fatigue, ataxia, dehydration or anorexia).

### Part 3: Tumor relapse

**Timing: 20 min (for step 5)**

**Timing: 30 min (for step 6)**

**Timing: 24–32 h (for step 7)**

**Timing: ∼24 h (for step 8)**

5. Detection of relapsed tumor by MRI.
  Tumor relapse and tumor volume in MPG mice after the irradiation are measured via MRI.
  a. Anesthetize the mouse by Ketamine/ Xylazine as described in step 2a.
  b. Visualize the tumor bearing brain by MRI using a GE MRI scanner (repetition, 3,450 ms; echo time, 159 ms; 12 slices at 0.8 mm per slice).
  c. Analyze the obtained MRI images using a T2-fast spin echo sequence.
6. Tumor Tissue Collection and Processing.
  Dissect the mouse brain and minimize disruption of brain tissues during collection.
  a. Euthanize mice according to the guidelines of the Institutional Animal Care and Use Committee (IACUC). MPG mouse can be euthanized by CO_2_ or by cervical dislocation following the routine procedure approved by the IACUC at Fox Chase Cancer Center.
  b. Decapitate the mouse head with a cut posterior from the ears using surgical scissors. Using the scissors, make a midline incision in the skin. Flip the skin over the eyes.
  c. Hold the mouse head with forceps, access the brain by inserting microdissection scissors horizontally into the foramen magnum and cutting straight between the eyes.
  d. Using forceps peel away the skull to expose the forebrain and cerebellum. Cut and remove the brain stem (anterior to the cerebellum) and forebrain as much as possible.
  e. Carefully rinse the cerebellum (containing MB tumor) with PBS.
7. Prepare Tumor Tissues for Immunohistochemistry.
  Brain and tumor tissues are processed for further immunohistochemistry analysis.
  a. Carefully place the cerebellum in a 15 mL tube filled with 4% PFA for fixation, and incubate overnight (∼12 h) at 4°C.
  b. Remove the cerebellum from 4% PFA solution and transfer to a new 15 mL tube filled with 30% sucrose for dehydration. Incubate at 4°C for 24 h or until the cerebellum sinks to the bottom of the tube.
  c. Embed the cerebellum in optimal cutting temperature compound (OCT compound), and freeze the block at −80°C overnight (∼12 h).
  d. Place the block in a −20°C cryostat for at least 1 h to equilibrate the tissue before proceeding to cryosectioning.
  e. Cut 8–12 μm thick frozen sections of the tumor-bearing cerebellum using a cryostat, and mount sections on microscope slides.
8. Immunofluorescent Staining.
  Brain tissues are harvested for immunofluorescence and microscopy analysis to detect changes in tumor cell proliferation, apoptosis, and astrocytic trans-differentiation patterns in relapsed tumor.
  a. Rinse the tumor slides with PBST.
  b. Carefully pipet 100 μL of 10% Normal Goat Serum (NGS) to cover entire section. Incubate for blocking at room temperature (20°C–25°C) for 20–30 min.
  c. Dilute the primary antibody(s) with 10% NGS, and pipet 50–100 μL of diluted primary antibody to cover the entire tissue section.
  d. Incubate the slides with the primary antibody (Anti-S100 β- 1:400, Anti-Cleaved-Caspase 3- 1:400, Anti-Ki67- 1:250) overnight (∼12 h) at 4°C, in the dark.
  e. Remove the primary antibody and wash the slides 3× with PBST.
  f. Prepare secondary antibody(s); dilute 1:500 in 10% NGS.
  g. Incubate the slides with secondary antibody for 2 h at room temperature (20°C–25°C), in the dark.
  h. Remove the secondary antibody and wash the slides 3× with PBS.
  i. Counterstain tumor tissue with DAPI to label cell nuclei.
  j. Mount coverslip on tumor slides using mounting medium.
  k. Image slides on a fluorescent microscope to analyze staining results.

## Expected outcomes

We optimized the dosage and age of tumor-bearing mice for irradiation, based on the survival after the irradiation (Table 1). Our results suggest that 0.5 Gy irradiation is a relatively safe dosage for Ptch1-deficient mice at 3 weeks of age.

**Table 1:** The survival of mice after irradiation

Dosage	Number of mice	Age of mice	Survival of mice
2 Gy	4	3 weeks	1/4
2 Gy	5	6 weeks	1/5
1.5 Gy	3	3 weeks	1/3
1.5 Gy	4	6 weeks	0/2
1 Gy	5	3 weeks	3/5
1 Gy	6	6 weeks	1/6
0.5 Gy	6	3 weeks	6/6
0.5 Gy	4	6 weeks	1/4

Justification:

Most MPG mice at 6 weeks of age died within 3 days following the irradiation at dosages ranging from 0.5–2 Gy, suggesting that irradiation is lethal for MPG mice at 6 weeks of age. Although a significant proportion of mice at 3 weeks of age succumbed to the irradiation at dosages of 1, 1.5, or 2 Gy, all mice (6/6) survived 0.5 Gy irradiation, suggesting that 0.5 Gy is a safe dosage for irradiating mice at 3 weeks of age.

**Figure 2:**
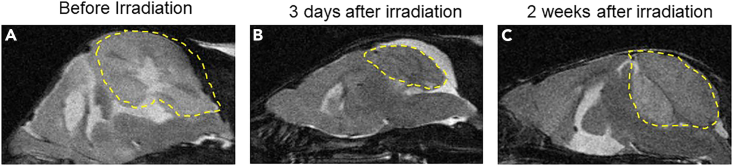
The change of tumor volume after irradiation (A–C) MRI brain images of a MPG tumor-bearing mouse at 3 weeks of age, (A) before irradiation, (B) 3 days following 0.5 Gy irradiation, and (C) 2 weeks following the irradiation, to show the establishment of MB tumor relapse after the irradiation.

Further MRI analyses revealed that irradiation at 0.5 Gy substantially reduced the volume of tumor in Ptch1-deficient mice (Figures 2A and 2B), and tumor volume significantly increased at 2 weeks following the irradiation (Figure 2C).

**Figure 3:**
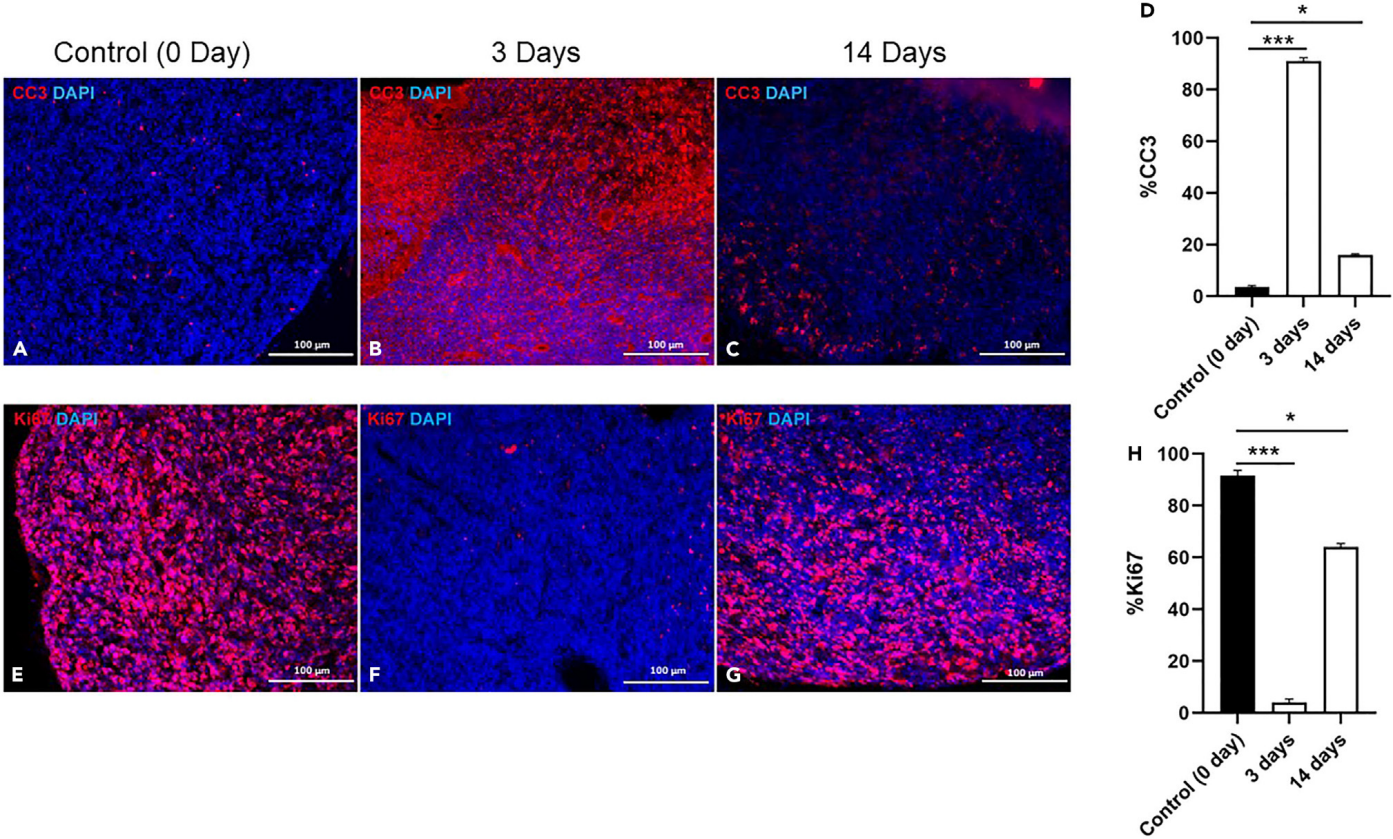
Evaluation of apoptosis in tumor tissue (A–D) Representative immunofluorescence images of cleaved caspase-3 (CC3) in tumor tissue from a MPG mouse at 3 weeks of age: (A) 0 day control (without irradiation); (B) 3 days following 0.5 Gy irradiation treatment; and (C) 2 weeks following 0.5 Gy irradiation; (D) The percentage of CC3 positive cells in tumor tissues was quantified. (E–H) Representative immunofluorescence images of Ki67 in tumor tissue from a MPG mouse of 3 weeks of age: (E) 0 day control (without irradiation); (F) 3 days following 0.5 Gy irradiation treatment; and (G) 2 weeks following 0.5 Gy irradiation; (H) The percentage of Ki67 positive cells in tumor tissues was quantified (∗, p < 0.05; ∗∗∗, p < 0.001). Scale bars represent 100 μm for all IF images.

Consistent with the tumor volume changes following the irradiation, extensive apoptosis was detected in tumor tissues within 3 days after the irradiation (Figures 3A and 3B). The percentage of apoptotic cells in tumor tissues at 2 weeks post-irradiation was reduced compared with that at 3 days following the irradiation, but was still increased compared with that in control tumor tissue (Figures 3C and 3D). Tumor cell proliferation was significantly inhibited by the irradiation (Figures 3E and 3F), as expected. However, tumor cells resumed their proliferation after 2 weeks following the irradiation (Figures 3G and 3H).

**Figure 4:**
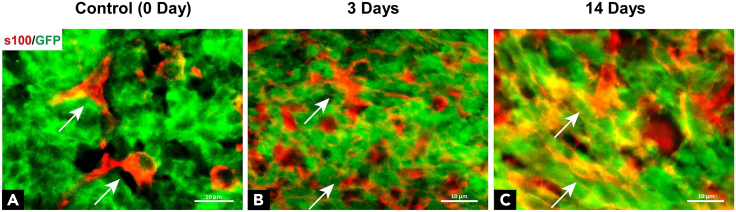
Evaluation of tumor cell trans-differentiation in tumor tissue (A–C) Representative immunofluorescence images of GFP and S100 β in tumor tissue from a MPG mouse at 3 weeks of age: (A) 0 day control (without irradiation); (B) 3 days following 0.5 Gy irradiation; and (C) 2 weeks following 0.5 Gy irradiation. Scale bars represent 10 μm for all IF images. Arrows point to astrocytes. Note that (C) majority of astrocytes were GFP positive, suggesting that astrocytes derive from tumor cells in the MPG mouse at 2 weeks following the irradiation.

Consistent with our previous report,^1^ astrocytes were found negative for GFP in tumor tissue from MPG mice (Figure 4A), suggesting that astrocytes and tumor cells were lineage-separated in primary MB. However, following the irradiation, an increased number of GFP positive astrocytes were detected in tumor tissues (Figure 4B). Until 2 weeks after the irradiation, majority of astrocytes were GFP positive (Figure 4C), indicating that most of astrocytes in the relapsed tumor originate from tumor cells.

## Quantification and statistical analysis

For quantification of immunofluorescent staining of brain tissue frozen sections, five fields were counted for each sample with each field containing approximately 2,000–2,500 cells. We reported the averages of these five fields. Statistical analysis was performed using SPSS Statistics software.

## Limitations

The optimal irradiation dosage and age of mice (tumor volume) for tumor relapse are determined using Math1-Cre/Ptch1^loxp/loxp^ mice in this study, which may not be applicable to other medulloblastoma mouse models such as NeuroD2-SmoA1 mice.

## Troubleshooting

### Problem 1

Mouse moves before or during irradiation treatment.

If the mouse moves in the chamber during irradiation exposure (Figure 1D), it may cause:

• The mouse will not receive the expected dosage of irradiation;
• The lead shield may not properly protect the mouse.

### Potential solution

• Mice may start to move during the irradiation, if too much time has passed after anesthetic solution injection. To avoid this, irradiate the mice within 30 min following the anesthetic solution injection.
• If needed, additional anesthetic solution (please refer to step 2a) may be applied to immobilize the mice during the irradiation.
  ○ Note that it is not recommended to administer an additional dosage of irradiation if mice move during the irradiation.
  ○ To achieve consistency and avoid outliers in the experiment, repeat if needed with a new animal.

### Problem 2

Regional difference in the distribution of cell apoptosis in tumor tissues after irradiation.

If the lead shield covers parts of cerebellar regions, it may block the exposure of tumor tissue to the irradiation, leading to uneven distribution of irradiation-induced apoptosis in tumor tissues.

### Potential solution

• Position the radiation area to the cerebellar region (tumor-bearing area) between the line aligning the mouse’s ears and the line aligning the skull base (please refer to Figure 1D). Make sure that the lead shield does not cover any part of the radiation area.

### Problem 3

Mice die following irradiation.

Post-irradiation, mice may present lethargic, decreased mobility and ataxia, which typically disappear after 3 days following the irradiation (step 4). However, too much irradiation exposure, excessive tumor burden before the irradiation, or lack of post-irradiation care may increase the mortality rate of irradiated mice.

### Potential solution

• Always irradiate the Ptch1-deficient MB-bearing mice with a dosage less than 1 Gy.
• Extensive cell apoptosis caused by irradiation in tumor tissues from Ptch1-deficient mice over 3–6 weeks of age, leads to a significant increase of intracranial pressure in mice after the irradiation, which often causes the death of irradiated mice.
  ○ It is recommended to use Ptch1-deficient mice at 3 weeks of age.
  ○ If needed, 20% Mannitol (1 g/kg of body weight) by intravenous (IV) injection^8^ may help to reduce the intracranial pressure in mice after the irradiation.
• A nutritionally fortified water gel can be put into the cage to aid the recovery of irradiated mice.

### Problem 4

Excessive background signal in irradiated tumor tissues after immunofluorescence.

Irradiation causes extensive cell death/apoptosis in tumor tissue, which may cause too much unspecific antibody staining.

### Potential solution

• Extend the blocking period to 2 h with 10% NGS before incubation of tumor tissues with the primary and secondary antibodies.
• If still experiencing unspecific staining, add 10% BSA to 10% NGS to block the tumor tissue before application of antibodies.

## Resource availability

### Lead contact

Further information and requests for resources and reagents should be directed to and will be fulfilled by the lead contact, Yijun Yang (Yijun.Yang@fccc.edu) or Zeng-Jie Yang (Zengjie.Yang@fccc.edu).

### Materials availability

No newly generated materials are associated with this protocol.

### Data and code availability

No datasets were generated for analysis in this protocol. No unique code was generated for this study.
